# Facilitators and Barriers to Developing a Research Program: A Focused Ethnography of New Tenure-Track PhD-Prepared Nursing Faculty

**DOI:** 10.1177/08445621241256702

**Published:** 2024-06-06

**Authors:** Winnifred Savard, Christy Raymond, Solina Richter, Joanne K. Olson, Pauline Paul

**Affiliations:** 1 Candidate at the Faculty of Nursing, 3158 University of Alberta, Edmonton, AB, Canada; 2 Dean of Nursing at the Faculty of Nursing, 3151 MacEwan University, Edmonton, AB, Canada; 3 Dean of Nursing at the College of Nursing, 7235 University of Saskatchewan, Saskatoon, SK, Canada

**Keywords:** PhD-prepared, tenure-track, nursing faculty, research program development, facilitators, barriers

## Abstract

**Background:**

Creating a research program is a critical requirement for new PhD-prepared tenure-track nursing faculty in Canada.

**Purpose:**

The purpose of this article is to present key findings of new faculty members focusing on facilitators and barriers to development of their research program.

**Method:**

We conducted focused ethnography research examining the experience of 17 new faculty members from across Canada.

**Results:**

The following themes were identified: teaching release, preparation from PhD program, intense feelings, supports and processes, mentoring, obtaining grants, and effects of the COVID-19 pandemic.

**Conclusions:**

Implications for practice include identifying ways to facilitate faculty retention as they develop their research program. This research will be of interest to deans of nursing and new faculty members.

The shortage of PhD-prepared faculty is a continuing concern for nursing education and one that makes faculty renewal imperative (Boamah et al., 2021). Recruitment and retention efforts have been recommended to reverse this trend and decrease the PhD-prepared faculty shortage (American Association of Colleges of Nursing, 2019; Canadian Association of Schools of Nursing, 2021). A review of the literature revealed that there is limited research regarding the experience of new PhD-prepared nursing academics (Savard et al., 2023). Knowing more about what facilitates the journey of these faculty is critical in the current context.

## Background and purpose

An unfunded, focused ethnography study was undertaken to better understand the experiences of new PhD-prepared nursing faculty in Canada. The purpose of this article is to present findings pertaining to developing research programs. We describe the method, key findings, and implications for practice. Findings about nursing academics’ transitions to the teaching role will be reported elsewhere.

In Canada, PhD in nursing programs are relatively new with the first students being admitted as special cases in the late 1980s, and programs formally beginning in the early 1990s (Patrick & McEwen, 2021). Currently, it is reported that 21 schools and universities offer PhD in Nursing programs (CASN, 2022). The total number of nursing faculty were reported to be 10915 members of whom 1639 members or 19.1% were in permanent (tenured or tenurable) contracts, and 50% of these were 50 or more years of age (CASN, 2022). Ninety-seven members or 5.9% of permanent faculty retired in 2021 and of those 97 people, 11 were under the age of 60 years of age (CASN, 2022). The number of early career faculty is not reported. Nursing seats for Entry-to-Practice (ETP) programs varied from province to province but the total Canadian intake of students for 2021 was indicated to be 16, 626 students (CASN, 2022) and admissions to PhD programs were 112 students (CASN, 2022). Despite slight increases in admissions to PhD programs and graduates from PhD programs, there was a predicted deficit of 2.4 times more faculty needed to be hired in 2022, with the average admission to PhD programs of 96 students (in the past 5 years), and only 65 PhD graduates in 2021 (CASN, 2022). These statistics emphasize the importance of increasing enrollment in PhD programs and enhancing retention strategies for future nurse faculty. As well, an increased understanding of the needs and beliefs of the new faculty is needed as the research about PhD-prepared faculty is sparse and it is not specifically known if new faculty are experiencing challenges with the tenure-track than previously (Savard et al., 2023).

Although granting of tenure is evaluated on tripartite components, developing a research program is the primary concern for new academics in nursing and other fields (Broome et al., 2019; Main et al., 2021; Singh et al., 2016; Stanfill et al., 2019). Lack of teaching release and preparation in grantsmanship were identified in studies as challenges to developing research programs (Cate et al., 2022; Singh et al., 2016). Having a child had been found to reduce research productivity, miss funding opportunities, and increase stress levels of female academics when compared with males (CohenMiller & Izekenova, 2022; James et al., 2021; Morgan et al., 2021). Interestingly, two of these studies did not include nursing academics and one did not identify the discipline of participants. Research indicates that new academics need clarity on tenure requirements, research mentors and supports, and would benefit from reduced teaching loads to establish their research program (Cate et al., 2022; Etzkorn & Braddock, 2020; Singh et al., 2016; Stamps et al., 2021; van Dongen et al., 2021). As we sought to have a deeper understanding of the experiences of new, PhD-prepared, nursing faculty, a focused ethnography research study was undertaken.

## Method and procedures

### Research design

A focused ethnography design was used to answer the research question: “What is it like to be a new (*new* defined as pre-tenured or within the first two years of tenure) tenure-track nursing faculty in Canada?” Focused ethnography is a research design that was developed from traditional ethnography. Traditional ethnography included three central components: observation in the natural setting of the participants, interviews with participants, and triangulation of data to explore the cultural beliefs and values of the population of interest. However, focused ethnography is well suited to study a smaller group's cultural beliefs/values in a specific setting or context and does not require conducting participant observations, requires less time than a traditional ethnographic study, and is an appropriate method for answering questions related to “what it is like to be…” (Roper & Shapira, 2000). As the authors were interested in exploring what it is like to be a new nursing faculty member, this cohort of faculty are a sub-set of the total new faculty population.

### Setting and sample

Initial Research Ethics Board (REB) approval was obtained from the University of Alberta, Pro00108151. Ethics and institutional approvals were obtained from 18 Canadian universities in total to increase the potential pool of participants. Participants volunteered from nine of these institutions. Eligibility criteria included: nursing faculty; PhD-prepared; and hired into a tenure-track position within the last six years. After approvals, administrative support individuals emailed an introductory study flyer to faculty members. Convenience sampling and snowball techniques enabled recruitment of 17 participants, and achievement of data saturation or the point at which no further new information was being reported.

### Data collection and analysis

Potential participants contacted the lead researcher by email and consent was obtained prior to data collection. Data were collected using semi-structured interviews, journaling, and the examination of public documents. The first author conducted recorded virtual interviews (55 to 80 min long) between March 2021 and April 2022, transcribed the recordings verbatim, and de-identified data. Field notes, during and after interviews, detailed researcher observations and impressions. Transcript clarifications with participants were conducted by email. The first author is not in an academic position but practices nursing and conducts research in a clinical setting. As the first author was a new qualitative researcher, the first author's supervisor (a senior academic and qualitative researcher) independently coded the first few interviews to provide inter-rater reliability. The other authors (also senior academics and experienced researchers) were involved in the review of the data analysis and development of the discussions.

Data collection and analysis took place concurrently. Roper and Shapira's (2000) steps for thematic analysis were utilized with the support of Quirkos*©* data management software. Constant comparison of data, memoing, reflexivity, and triangulation enhanced rigor (Creswell & Creswell, 2018). Public documents i.e., tenure criteria documents, research release documents, and collective agreements were retrieved from public university websites (University of Alberta Faculty of Nursing, 2018; University of Toronto, 2023; York University, 2017). To reduce the potential for identification of participants, the cited documents are examples and not the comprehensive list of documents retrieved for analysis. The public documents were reviewed and analyzed by content and compared between universities (Bowen, 2009) and provided triangulation of data for the research. The data from these documents confirmed what participants expressed about tenure criteria and expectations ambiguity and workload adjustments. Trustworthiness was established using Lincoln and Guba's (1985) criteria for qualitative research.

## Results

Participant characteristics are presented in Table 1 (below). The participants were recruited from universities in several provinces and regions of Canada. In Canada, nursing faculty generally work in unionized institutions that receive public funding from the provinces or territories as well as student tuition fees. Canadian admissions to ETP programs in 2021 were 16, 626 and admissions to PhD programs were 112 (CASN, 2022), and 65 students graduated from PhD programs in 2021 (CASN, 2022). These statistics indicate increasing admissions to ETP and slightly higher admissions to PhD programs.

**Table 1.** Participant Characteristics.

Participant ID	Self-identified gender	Relationship status	Age category (years)	# Years on tenure-track	Academic experience (years)	Clinical experience (years)	Post-doc Y/N	Institution focus	Young children < 13 years old
1	F	Partner	46–55	0.5	4	16–20	Y	teaching	Y
2	F	Partner	36–45	3	5	5–10	Y	research	Y
3	F	Partner	36–45	2	5	5–10	Y	research	Y
4	F	Partner	36–45	5	6	5–10	Y	research	Y
5	F	Partner	36–45	6	7+	<5	Y	research	Y
6	M	Partner	36–45	3	7+	21–25	Y	research	N
7	F	Partner	36–45	2	7+	21–25	N	research	N
8	F	Partner	46–55	1	7+	11–15	N	teaching	N
9	M	Partner	56+	1	7+	16–20	N	research	N
10	F	Partner	25–35	2	4	<5	N	teaching	N
11	F	Partner	25–35	2	4	<5	Y	research	N
12	F	Partner	36–45	3.5	5	<5	Y	research	Y
13	F	Single	36–45	4	7+	11–15	Y	teaching	N
14	F	Partner	36–45	3	5	<5	Y	research	Y
15	F	Partner	56+	2	7+	16–20	N	research	N
16	F	Partner	56+	.25	7+	5–10	N	teaching	N
17	F	Partner	36–45	.25	5	5–10	N	teaching	N

Seven themes were elucidated concerning research program development: teaching release, preparation from PhD education, mentoring, supports and processes, obtaining grants, negative feelings, and effects of the CoVID-19 pandemic. Perceptions and experiences of the participant influenced whether the theme was a barrier or facilitator.

### Teaching release

During interviews, participants indicated teaching release was extremely helpful when developing a research program. Participant 6 explained: “Due to my research heavy portfolio, I could negotiate more teaching release.” Participant 2 indicated: “I negotiated one course release per term for the first year, which was extremely helpful because the hardest part was setting up a research program. That was the biggest surprise for me about being an academic.”

Participants indicated not having teaching release was problematic. Participant 9 explained: “I was not supposed to teach the first semester, but that was retracted. The only time for research was during my summer vacation.” Similarly, Participant 1 indicated: “I had no release for research. I have a paper waiting to be published for three years.” Another, Participant 5, shared: “I was told I would not get any course release until I received a grant, which I thought was very backward, but I wanted a job. So, I agreed.” Participant 15 said: “The stressful part was creating that program of research without release time.” New faculty needed and desired a reduction in their teaching load to have time to develop their program of research and produce scholarly work.

### Preparation from PhD education

Several participants considered their research preparation inadequate, while others felt prepared to establish a research program. Participant 3 commiserated: “There's information not taught in any graduate program, like self-discipline. How do you organize a lab? How do you negotiate?” Participant 15 wanted education about “best practices on grant writing, publication, and those things that you may not get in your PhD.” Participant 10 reflected: “I studied at a research-intensive university. I feel confident with my abilities to tackle research and write grants.”

Ten participants had completed post-doctoral fellowships and felt more competent and prepared to build a research program. Participant 11 explained: “That post-doc allowed me to conduct applied research*.”* Participant 6 recollected: “A post-doc was critical to my success. Many of the things you're required to do as a researcher, you won't learn in a PhD.” Participant 2 commented: “It was a shocking immersion into research. I suddenly realized that research was a lot more detailed.” Findings from our study suggest that PhD education programs provide insufficient instruction in the key areas such as grant writing, building a research team and program, that could ease the stress of performing these tasks independently.

### Mentoring

Several participants discussed inadequate mentoring. Participant 16 said: “I need some mentorship, but it seems like it's figure it out yourself. That is the way it has always been.” Participant 9 explained: “I finally got a research mentor but other than being the same sexual orientation as me, there's no connection between us.” Alternatively, participant 17 indicated: “I have exceptional mentorship. I had meetings about my research in their homes. The work culture of my Faculty is impeccable.”

Participant 8 discussed mixed mentoring:

I prefer formal and informal mentors, who tell you what is really going on, and you can feel safe talking about your fears. Due to these supports, I was successful enough with grants and publications to be on track with tenure.

Participant 11 engaged in peer mentorship: “I didn’t know how to manage a research team or human resources. I would be floating in the abyss if I didn't have a colleague to share that with.” The participants valued mentorship and expected to have mentorship as a part of their early career experience.

### Supports and processes

All participants commented about start-up funds available for new faculty members. Participant 17 stated: “The faculty calculate a lot; funds I got were based on a very fair calculation.” Participant 7 was exuberant: “I got the highest faculty start-up funds that the university ever had!” Likewise, Participant 14 stated, “I got start-up funds, but I applied for [a grant] and got another smaller sum.” Participant 5 wryly stated: “I was told to apply for $12,000. The faculty (conducting that process) helped me. I got four times that amount!” Participant 4 argued funding was insufficient: “It was not enough to start a bigger project.” Participant 8 wistfully stated: “Start-up funds would have helped [second tenure-track]. Even though you're a little more established, you're still starting up.”

Some voiced concern over a lack of institutional supports and safety. Participant 5 stated: “My faculty has been non-existent. Zero interest in helping, it's your problem, go figure it out” and they felt unsafe: “I didn't feel safe because I was terrified of the repercussions [to my tenure progress] by asking for assistance.” Participant 9 was disillusioned: “Many challenges I experienced were due to unfulfilled promises.” Participant 7 experienced disparagement during a required orientation meeting with research administrators: “They pushed me this list and said, we’re going through all the criteria that you need to meet as a tenure-track faculty. You’ll have five minutes at the end to talk*.*” Participant 7 elaborated: *“*I was being positioned. I did not like it! I know this isn't a safe place for me to go to if I have concerns.”

Bureaucratic institutional processes had negative effects. Participant 7 experienced challenges spending funds: “I didn't know about an approved vendor list, now, I'm embroiled in bureaucratic processes*.”* Participants 8 and 9 had relocated during the tenure-track period. Participant 9 bitterly expressed: “[the faculty] gave no recognition, I was starting at square zero” and Participant 8 ruefully said: “I had to restart tenure.” Participant 7 highlighted reduced transparency about research processes: “The institution should have algorithms for this. It goes through ethics. Then, these are the other components.” Participant 8 stated: “I knew I needed ethics at the university, but they took longer than I expected.”

As suggested by the participants, institutional supports and processes are inconsistent. Some institutions provided start-up funds for all new faculty while others did not. Bureaucratic processes increased stress and disillusionment with support desired by new faculty.

### Obtaining grants

All participants identified grantsmanship as essential for progression and tenure. Many reported difficulty obtaining grants. Participant 6 illuminated: “Money is scarce. It's a very tough game right now.” Participant 12 shared: “I compete against myself [for grants]. Anything I do that improves grants [as co-investigator] decreases my chances of getting them as a PI.” Participant 4 stated: “It's hard establishing a program of research. It is extremely competitive and subjective.” Participant 2 inferred: “People providing the grants even indicate it is subjective.” In the current competitive climate, learning how to develop, write, and obtain grants is a core aspect to developing a successful research program and participants were highly aware of this challenge in their tenure-track journey.

### Negative feelings

Participants expressed feelings of self-doubt, failure, and stress. Participant 2 stated: “You question yourself [constantly] about the quality and value of your work.” and expressed perceived discrimination: “You won a grant [as the “token” immigrant].” Participant 4 reflected: “It's exceedingly stressful to cope with failure, you feel you're worthless!” Participant 5 recounted: “You’re not good at teaching, research, or anything!”

Many participants expressed confusion about tenure criteria. Participant 13 summarized: “Last year, the faculty started trying to quantify criteria for tenure. There was quite an argument! How do you quantify what is needed? Currently, there isn't clarity about what is required.” Participant 11 felt stressed about tenure criteria: “The minimum requirements are vague. I struggle! Am I going to be compared to other faculty's output, or am I just evaluated on these minimum criteria?” Participant 14 articulated: “I don’t know how my scholarship will be judged!” Participant 12 shared: “If I focus too much on the outcomes that might be valued, I find it is soul crushing!”

All participants indicated feelings of constant pressure, competition, and intensity of work. Participant 8 articulated: “The pressure you feel to succeed and the “grunt “work required is really intense.” Participant 16 indicated “I feel this constant pressure.” The competitive environment and internal competitiveness were acknowledged as “an inherent part of academia, and it's within yourself. Really, we're (faculty) all type A personalities.” (P2) and “they (mentors) were still competitive. I even feel it (competition) in myself, I just think it's a competitive world.” (P8)

### COVID-19 pandemic

The COVID-19 pandemic halted most participants’ productivity but two benefited. Participant 10 reflected: “During a pandemic, I won’t have as many papers as previous new faculty.” Participant 6 stated: “I was even busier! I got two grants during COVID.” Participant 7's research flourished “(COVID-19) opened another research opportunity and propelled my research.” However, participants acknowledged institutions provided a year extension to their tenure or promotion review.

## Discussion

Based on this research, the identified themes of mentorship, teaching release, PhD preparation, supports and processes, obtaining grants, negative feelings, and COVID-19 pandemic effects were facilitators or barriers to developing a research program. Our findings illuminated mentoring, supports (i.e., start-up funds, teaching release) and clear processes (faculty or institutional) as facilitators (or barriers, if absent) to research program development. As in other studies, (Boamah et al., 2021; Sherman et al., 2023; Singh et al., 2016; van Dongen et al., 2021) mentorship and teaching release were deemed critical for developing a research program but were inconsistently offered by institutions. Teaching release time is an offered or negotiated reduction in teaching load requirements during the tenure-track period (Boamah et al., 2021; Singh et al., 2016; University of Alberta Faculty of Nursing, 2018; University of Toronto, 2023; York University, 2017). Typical workload requirements reported by our participants were 40% research, 20% service, and 40% teaching and this was comparative to those reported in public documents (University of Alberta, 2020). Furthermore, Bittner and O’Connor (2012) indicated that lack of teaching release time for research development was identified as a barrier to job satisfaction and retention.

Our findings also indicated that participants were stressed. Formalized mentoring programs have been found to reduce issues of transition into academia, attrition, and stress (Etzkorn & Braddock, 2020; Volkert, 2021) but other research indicates that faculty shortages continue, and new faculty's experiences are challenging amidst this competitive landscape (Singh et al., 2016; Stamps et al., 2021). Contrary to other research (CohenMiller & Izekenova, 2022; James et al., 2021; Morgan et al., 2021) and requiring further investigation, participants with young children did not report impedance to research development. A new finding was the internal competitiveness of the participants, and the effect of this trait needs further investigation.

Barriers identified by participants were negative feelings of un-preparedness, not feeling safe, or inadequacy; confusion regarding tenure criteria; and intense pressure to obtain grants. Our findings indicate that faculty need clearer tenure expectations and standardized support practices. Our results emphasized the necessity for multiple institutional supports such as formal mentoring, teaching release time, and start-up funding, which is echoed in other research (Boamah et al., 2021; Singh et al., 2016). Although more confident, even participants with postdoctoral education suggested that more faculty development is needed (Main et al., 2021; Shaaban et al., 2022; van Dongen et al., 2021). The pandemic was both a facilitator and barrier for participants.

### Implications for practice

Mentorship is crucial for new faculty establishing research programs. Teaching release time and start-up funds are helpful for all new tenure-track faculty. The hiring investment needs to incorporate the support of tenure-track faculty and clear guidelines for tenure and research processes are essential. Education about how to apply for academic positions and negotiate supports is recommended. Since obtaining research funding is essential for achieving tenure, more instruction about developing a research program and practical information about general university structures that support research should be included in PhD programs.

### Areas for future research

There are several areas related to research program development needing further exploration. Areas include childbearing/rearing academics’ experiences with research development; COVID 19 effects on achieving promotion; the influence of internal competition; and diversity, equity, and inclusivity concerns.

### Strengths/limitations

A strength of this study is that it included participants from nine institutions from across Canada rather than all participants coming from one institution or province. This increased the transferability to other settings as participants from various regions and universities in Canada provided rich data**.**

A limitation was that data collection took place during the pandemic, and COVID-19 may have influenced perceptions. Another limitation was the expectation of some institutions that a faculty member from their institution be included to apply for ethics approval. As this study was conducted as part of the first author's dissertation work, it would have been unrealistic to add numerous other authors to this work. This limited access to some Canadian institutions and potential participants.

## Conclusion

Participants in this study implied dissatisfaction from unfulfilled expectations could lead to attrition. As these feelings are not well portrayed in the research literature, our research contributes to the body of literature about tenure-track experiences in Canadian academic culture. Implications for practice include the need for enhanced supports for new faculty. Increased satisfaction with academic roles may increase graduate enrolment, faculty recruitment, and retention.

## References

[bibr1-08445621241256702] American Association of Colleges of Nursing (2019). Special survey on vacant faculty positions for academic year 2018-20191. https://www.aacnnursing.org/Portals/42/News/Surveys-Data/vacancy08.pdf

[bibr2-08445621241256702] Bittner N. P. O’Connor M. (2012). Assessment of the impact of teaching demands on research productivity among doctoral nursing program faculty. Journal of Professional Nursing, 33(4), 251–254. https://doi.org/https://www.aacnnursing.org/Portals/42/News/Factsheets/Faculty-Shortage-Factsheet.pdf 10.1016/j.profnurs.2015.06.011 27216126

[bibr3-08445621241256702] Boamah S. A. Callen M. Cruz E. (2021). Nursing faculty shortage in Canada: A scoping review of contributing factors. Nursing Outlook, 69(4), 574–588. 10.1016/J.OUTLOOK.2021.01.018 33707118

[bibr4-08445621241256702] Bowen G. A. (2009). Document analysis as a qualitative research method. Qualitative Research Journal, 9(2), 27–40. 10.3316/QRJ0902027

[bibr5-08445621241256702] Broome M. E. Oermann M. H. Douglas C. E. Simmons D. F. Woodward A. (2019). Publication productivity of nursing faculty in selected schools of nursing across the United States. Journal of Nursing Scholarship, 51(3), 346–355. 10.1111/j.nu.12463 30762935

[bibr6-08445621241256702] Canadian Association of Schools of Nursing (2021). Registered nurses’ education in Canada statistics. 2019-2020. https://www.casn.ca/2021/12/registered-nurses-education-in-canada-statistics-2019-2020/

[bibr7-08445621241256702] Canadian Association of Schools of Nursing (2022). Registered Nurses education in Canada statistics 2020–2021. Registered Nurse Workforce, Canadian Production: Potential New Supply. Canadian Association of Schools of Nursing. 2020-2021-CASN-Student-Faculty-Survey-Report.pdf

[bibr8-08445621241256702] Cate L. Ward L. W. M. Ford K. S. (2022). Strategic ambiguity: How pre-tenure faculty negotiate the hidden rules of academia. Innovative Higher Education, 47(5), 795–812. 10.1007/s10755-022-09604-x 35502224 PMC9046712

[bibr9-08445621241256702] CohenMiller A. Izekenova Z. (2022). Motherhood in academia during the COVID-19 pandemic: An international online photovoice study addressing issues of equity and inclusion in higher education. Innovative Higher Education, 47(5), 813–835. 10.1007/s10755-022-09605-w 35615725 PMC9123391

[bibr10-08445621241256702] Creswell J. W. Creswell J. D. (2018). Research design: Qualitative, quantitative, and mixed methods approaches. Sage Publications, Inc.

[bibr11-08445621241256702] Etzkorn K. B. Braddock A. (2020). Are you my mentor? A study of faculty mentoring relationships in US higher education and the implications for tenure. International Journal of Mentoring and Coaching in Education, 9(3), 221–237. 10.1108/IJMCE-08-2019-0083

[bibr12-08445621241256702] James Y. Bourgeault I. Gaudet S. Bujkai M. (2021). Care and academic motherhood: Challenges for research and tenure in the Canadian university. Canadian Journal of Higher Education/Revue Canadienne D’enseignement Supérieur, 51(4), 86–99. 10.47678/cjhe.v51i4.189061

[bibr13-08445621241256702] Lincoln Y. S. Guba E. (1985). Naturalistic Inquiry. Sage.

[bibr14-08445621241256702] Main J. B. Wang Y. Tan L. (2021). The career outlook of engineering PhDs: Influence of postdoctoral research positions on early career salaries and the attainment of tenure-track faculty positions. Journal of Engineering Education, 110(4), 977–1002. 10.1002/jee.20416

[bibr15-08445621241256702] Morgan A. C. Way S. F. Hoefer M. J. D. Larremore D. B. Galesic M. Clauset A. (2021). The unequal impact of parenthood in academia. Science Advances, 7(9), 1–8. 10.1126/sciadv.abd1996 PMC7904257 33627417

[bibr16-08445621241256702] Patrick J. L. McEwen A. E. (2021). The growth of graduate education in nursing. In McCleary L. McParland T. (Eds.), Ross-Kerr and wood’s Canadian nursing issues & perspectives ((6th ed, pp. 419–433). Elsevier.

[bibr17-08445621241256702] Roper J. M. Shapira J. (2000). Ethnography in Nursing Research. Sage Publications Inc.

[bibr18-08445621241256702] Savard W. Paul P. Raymond C. Richter S. Olson J. (2023). Experiences of new tenure-track PhD-prepared faculty: A scoping review. International Journal of Nursing Education Scholarship, 40(1), 1–14. 10.1515/ijnes.2022-0025 37167279

[bibr19-08445621241256702] Shaaban C. E. Dennis T. L. Gabrielson S. Miller L. J. Zellers D. F. Levine A. S. Rosana C. (2022). Retention, mobility, and successful transition to independence of health sciences postdocs. PLoS ONE, 17(11), 1–12. 10.1371/journal.pone.0276389 PMC9624420 36318574

[bibr20-08445621241256702] Sherman J. Kalvas L. B. Schlegal E. C. (2023). Navigating the turbulent seas: Experiences of peer mentorship on the journey to becoming a nurse scholar. Nurse Education Today, 121, 1–7. 10.1016/j.nedt.2022.105694 PMC9916728 36535122

[bibr21-08445621241256702] Singh M. D. Patrick L. Pilkington B. (2016). An exploration of the pre-tenure and tenure process experiences of Canadian nursing faculty. Quality Advancement in Nursing Education, 2(2), Art. 2. 10.17483/2368-6669.1062

[bibr22-08445621241256702] Stamps A. Cockerell K. Opton L. (2021). A modern take on facilitating transition into the academic nurse educator role. Teaching and Learning in Nursing, 16(1), 92–94. 10.1016/j/teln.2020.04.002

[bibr23-08445621241256702] Stanfill A. Aycok D. Dionne-Odom J. N. Rosa W. E. (2019). Strategies and resources for increasing the PhD pipeline and producing independent nurse scientists. Journal of Nursing Scholarship, 51(6), 717–726. https://doi.org/10.1111.jnu.12524 31697044 10.1111/jnu.12524

[bibr25-08445621241256702] University of Alberta (2020). Assistant/Associate or Full professor, tenure-track faculty of nursing. https://www.careers.ualberta.ca/Competition/A102349194D1/-assistant-associate_or_full_professor_tenure-track_faculty_of_nursing-university_of_alberta-toronto/

[bibr24-08445621241256702] University of Alberta Faculty of Nursing (2018). Faculty of Nursing research allowance guidelines and application (pp. 1–4). faculty-of-nursing-research-allowance-guidelines—general.pdf (ualberta.ca).

[bibr26-08445621241256702] University of Toronto (2023). Guidelines: Teaching releases to support research and scholarship. TeachingReleaseGuidelines_November2023.pdf (utoronto.ca).

[bibr27-08445621241256702] van Dongen L. Cardiff S. Kluijtmans M. Schoonhoven L. Hamers J. P. H. Schuurmans M. J. Hafsteinsdóttir T. B. (2021). Developing leadership in postdoctoral nurses: A longitudinal mixed-methods study. Nursing Outlook, 69(4), 550–564. 10.1016/j.outlook.2021.01.014 33750611

[bibr28-08445621241256702] Volkert D. (2021). Development and implantation of a strong mentoring program to increase scholarly productivity and support nurse faculty retention. Nursing Education Perspectives, 42(6), e77–e78. 10.1097/01.NEP.0000000000000837 34085997

[bibr29-08445621241256702] York University (2017). Teaching workload policy for full-time faculty members. Microsoft Word – SOSC 201718 teaching load document.doc (yorku.ca).

